# Cross-Talking of Pathway-Specific Regulators in Glycopeptide Antibiotics (Teicoplanin and A40926) Production

**DOI:** 10.3390/antibiotics12040641

**Published:** 2023-03-24

**Authors:** Andrés Andreo-Vidal, Oleksandr Yushchuk, Flavia Marinelli, Elisa Binda

**Affiliations:** 1 Department of Biotechnology and Life Sciences, University of Insubria, via J. H. Dunant 3, 21100 Varese, Italy; 2 Department of Genetics and Biotechnology, Ivan Franko National University of Lviv, 79005 Lviv, Ukraine

**Keywords:** *Actinoplanes teichomyceticus*, *Nonomuraea gerenzanensis*, teicoplanin, A40926, dalbavancin, glycopeptide antibiotics, biosynthesis regulation

## Abstract

Teicoplanin and A40926 (natural precursor of dalbavancin) are clinically relevant glycopeptide antibiotics (GPAs) produced by *Actinoplanes teichomyceticus* NRRL B-16726 and *Nonomuraea gerenzanensis* ATCC 39727. Their biosynthetic enzymes are coded within large biosynthetic gene clusters (BGCs), named *tei* for teicoplanin and *dbv* for A40926, whose expression is strictly regulated by pathway-specific transcriptional regulators (PSRs), coded by cluster-situated regulatory genes (CSRGs). Herein, we investigated the “cross-talk” between the CSRGs from *tei* and *dbv*, through the analysis of GPA production levels in *A. teichomyceticus* and *N. gerenzanensis* strains, with knockouts of CSRGs cross-complemented by the expression of heterologous CSRGs. We demonstrated that Tei15* and Dbv4 StrR-like PSRs, although orthologous, were not completely interchangeable: tei15* and *dbv4* were only partially able or unable to cross-complement *N. gerenzanensis* knocked out in *dbv4* and *A*. *teichomyceticus* knocked out in tei15*, implying that the DNA-binding properties of these PSRs are more different *in vivo* than it was believed before. At the same time, the unrelated LuxR-like PSRs Tei16* and Dbv3 were able to cross-complement corresponding *N. gerenzanensis* knocked out in *dbv3* and *A. teichomyceticus* knocked out in tei16*. Moreover, the heterologous expression of *dbv3* in *A. teichomyceticus* led to a significant increase in teicoplanin production. Although the molecular background of these events merits further investigations, our results contribute to a deeper understanding of GPA biosynthesis regulation and offer novel biotechnological tools to improve their production.

## 1. Introduction

Actinobacteria produce more than two-thirds of antibiotics used in medicine and agriculture, as well as other specialized metabolites [1]. One example of these valuable compounds are glycopeptide antibiotics (GPAs) which are last-resort drugs against multidrug-resistant Gram-positive pathogens such as staphylococci, enterococci, and *Clostridioides difficile* [2,3]. Natural GPAs are produced by fermentation of filamentous actinobacteria mainly from the genera *Amycolatopsis, Actinoplanes,* and *Nonomuraea* [4]. They are divided into five types according to their structure; among them, types I-IV (also known as dalbaheptides) are clinically relevant [4,5]. Clinically important GPAs include first-generation vancomycin and teicoplanin [6], which are produced by *Amycolatopsis orientalis* strains [7] and *Actinoplanes teichomyceticus* NRRL B-16726 [8], respectively, by fermentation processes that were developed and have been described in the past [9,10,11,12,13,14]. Vancomycin was introduced in clinics first (in 1958) [15], followed by teicoplanin (in 1988 in Europe and in 1998 in Japan) [16,17]. Second-generation GPAs are semisynthetic molecules, approved for therapeutic use in the last decade, chemically deriving either from the natural vancomycin-like molecules produced by fermentation (telavancin and oritavancin) [18] or from A40926 (dalbavancin) [19]. The latter natural precursor of dalbavancin—A40926—is biosynthesized by the uncommon actinobacterium *Nonomuraea gerenzanensis* ATCC 39727 [20]. The renewed interest for these antibiotics and their newest developments, highlighting the importance of developing next-generation semisynthetic GPAs with enhanced antibacterial activities and improved safety profiles, have been recently stated in different reviews [21,22,23,24,25]. Recent efforts have also aimed at developing targeted therapies, and advances have been made in extending the activity of GPAs toward Gram-negative organisms [21,22,23,24,25].

As for the majority of specialized metabolites, GPA biosynthetic enzymes are coded within large biosynthetic gene clusters (BGCs) [21,22]. Similarly to other antibiotic biosynthetic pathways [26], the expression of GPA BGCs is strictly regulated by pathway-specific transcriptional regulators (PSRs), coded by cluster-situated regulatory genes (CSRGs). It is likely that the expression of these CSRGs is by turn controlled by pleiotropic regulators of a higher rank, although the knowledge of their role is still very limited [27,28]. All the described GPA BGCs contain at least one CSRG, coding for a StrR-like [29] transcriptional regulator [30]. An additional CSRG could be present in GPA BGCs [30], coding for a large ATP-binding regulator belonging to the LuxR family [31,32]. The roles of CSRGs have been investigated in *Amycolatopsis balhimycina* DSM 5908 [33], *Amycolatopsis japonicum* MG417-CF17 [12], *Amycolatopsis* sp. TNS106 [13], *Amycolatopsis orientalis* NCPC 2-48 [34], *A. teichomyceticus* NRRL B-16726 [30,35], and *N. gerenzanensis* ATCC 39727 [36,37,38], producing balhimycin, ristocetin (both MG417-CF17 and TNS106), norvancomycin, teicoplanin, and A40926, respectively. The best-studied cases are those of *Am. balhimycina* DSM 5908, *A. teichomyceticus* NRRL B-16726, and *N. gerenzanensis* ATCC 39727. The expression of balhimycin BGC—*bal* [39]—is controlled by a single StrR-like PSR, named Bbr [33]. Teicoplanin BGC (called *tei*) encodes two PSRs: Tei15* (StrR-like) and Tei16* (LuxR-like) [40,41], while A40926 BGC (called *dbv*) also codes for two PSRs: Dbv3 (LuxR-like) and Dbv4 (StrR-like) [42].

The transcriptional control of BGCs with only one CSRG seems to be rather straightforward and is well represented by the model involving Bbr, which singularly controls the expression of the majority of *bal* genes and operons [33]. On the contrary, the regulatory mechanisms of the GPA BGCs (as *tei* and *dbv*) carrying two different CSRGs are more intriguing. Previous works reported that either in *A. teichomyceticus* or in *N. gerenzanensis,* the expression of both CSRGs is essential for GPA biosynthesis, since the knockout of each of them completely abolished antibiotic production [35,37]. In addition, the overexpression of tei15*, tei16*, *dbv3*, and *dbv4* significantly improved GPA production in the homologous producers [35,37,38]. Nevertheless, the logic behind the functions of the PSRs of *tei* and *dbv* is quite different. Notably, in *A. teichomyceticus,* the StrR-like regulator—Tei15*—was shown to directly control the expression of the majority of the *tei* genes (5) and operons (5), coding for NRPS, for tailoring enzymes, and for enzymes of non-proteinogenic amino acids biosynthesis [30]. At the same time, tei15* itself seemed to be the single target of LuxR-like Tei16* [30]. On the contrary, in *N. gerenzanensis,* StrR-like PSR Dbv4 was responsible for the activation of only two operons: *dbv14–8* (coding for cross-linking monooxygenases, halogenase, glycosyltransferase, and acyltransferase) and *dbv30–35* (coding for the l-3,5-dihydroxyphenylglycine biosynthesis enzymes) [37]. The other operons (5) and genes (2) of *dbv* appeared to be under the control of the LuxR-like PSR Dbv3 [37]. Hence, the *tei* expression seems to be controlled by Tei15* and Tei16* in a hierarchical fashion, while Dbv3 and Dbv4 possess two separate regulons within *dbv*.

The DNA-binding properties of StrR-like PSRs coded in GPA BGCs have been extensively investigated and seem to be quite similar [33,35,36]. On the other hand, the exact mechanisms of action of LuxR-like PSRs from *A. teichomyceticus* (Tei16*) and *N. gerenzanensis* (Dbv3) remain obscure. In particular, the analysis of Tei16* DNA-binding properties in vitro failed to reveal any target within *tei* [35], while the DNA binding features of Dbv3 have not been investigated yet.

A recent work reconstructing the overall phylogeny of StrR-like PSRs coded within GPA BGCs demonstrated that Dbv4 and Tei15* belong to two separate clades of the phylogenetic tree, while Dbv3 and Tei16* are likely non-orthologous, having emerged in the corresponding BCGs independently. Notably, Dbv4 was found to be closely related to Bbr and to Ajr (the StrR-like PSR encoded within ristocetin BGC from *Am. japonicum* MG417-CF17), and indeed all these PSRs showed a high degree of interchangeability in regulating cognate GPA BGCs [12,37]. However, the degree of interchangeability of less related PSRs, such as the abovementioned Dbv4 and Tei15*, as well as Dbv3 and Tei16*, has not been investigated yet.

In the current work, we investigated the “cross-talk” between the CSRGs from *tei* and *dbv* BGCs. We demonstrated that tei15* and *dbv4* are not completely interchangeable, implying that the DNA-binding properties of phylogenetically distant StrR-like PSRs are more different *in vivo* than was supposed. At the same time, surprisingly, unrelated LuxR-like PSRs were able to cross-complement corresponding mutants of *A. teichomyceticus* and *N. gerenzanensis.* Moreover, the overexpression of *dbv3* in *A. teichomyceticus* led to a significant increase in teicoplanin production.

## 2. Results

### 2.1. Generation and Complementation of dbv3 and dbv4 N. gerenzanensis Knockout Mutants

Previously, we reported that the knockouts of tei15* and tei16* CSRGs in *A. teichomyceticus* completely abolished the production of teicoplanin, while the re-introduction of wild type alleles restored antibiotic biosynthesis [35]. In this work, we knocked out *dbv3* and *dbv4,* replacing them with the spectinomycin/streptomycin resistance cassette *oriT-aadA* by using the λ-Red-mediated recombineering approach [43]. Although other authors have previously reported on the generation of *dbv4* and *dbv3* knockout mutants where the apramycin resistance cassette (*oriT-aac(3)IV*) replaced target genes [37], for the current work, we required apramycin-sensitive mutants. Our strategy for performing knockouts of *dbv3* and *dbv4* is described in the Materials and Methods (Section 4) and is reported in detail in Figure S1 (Supplementary Materials).

**Figure 1:**
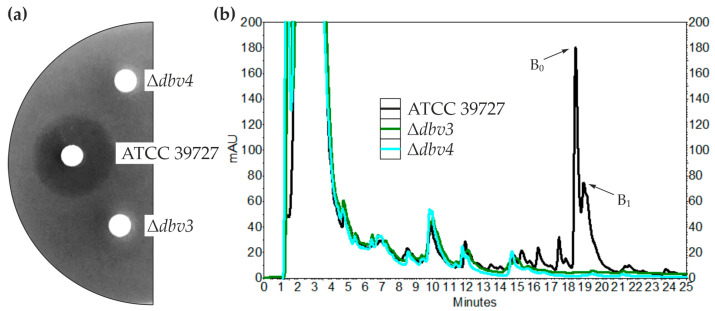
A40926 production is abolished in *N. gerenzanensis* Δ*dbv3* and Δ*dbv4* mutants. (**a**) *B. subtilis* HB0933 growth inhibition assay (MHA medium), demonstrating that *N. gerenzanensis* Δ*dbv3* and Δ*dbv4* knockout strains did not show any antimicrobial activity. To prepare the assay, culture broth samples were collected after 144 h cultivation of *N. gerenzanensis* ATCC 39727, Δ*dbv3*, and Δ*dbv4* strains in FM2 liquid medium. A40926 was extracted as reported in Materials and Methods. In total, 50 μL of the extracts were loaded onto 6 mm paper disks. (**b**) HPLC analysis of the same extracts confirmed that *N. gerenzanensis* Δ*dbv3* and Δ*dbv4* did not produce any A40926 in FM2 medium; *N. gerenzanensis* ATCC 39727 chromatographic profile is shown in black, *N. gerenzanensis* Δ*dbv3* in green, while *N. gerenzanensis* Δ*dbv4* in blue. Two main A40926 peaks could be distinguished in the *N. gerenzanensis* ATCC 39727 chromatographic profile: B_0_ and B_1_ are indicated, respectively, by black arrows; they differ for the fatty acid moiety (*iso*-C12:0 in B_0_, and *n*-C12:0 in B_1_ [44]). These two peaks are absent in the chromatograms from Δ*dbv3* and Δ*dbv4* mutants (for the chromatogram of commercial A40926 standard, please refer to Figure S2a).

Obtained mutants—*N. gerenzanensis* Δ*dbv3* and Δ*dbv4* (Table 1)—were cultivated in the FM2 medium (optimized for high-level A40926 production in *N. gerenzanensis* [11]). Both mutants were not able to produce A40926, as confirmed using a *B. subtilis* HB0933 growth inhibition assay (Figure 1a) and HPLC analysis (Figure 1b).

To complement *N. gerenzanensis* Δ*dbv3* and Δ*dbv4,* two different platforms for the *dbv3* and *dbv4* expression were tested, being either pSET152A [45] or pIJ12551 [46] derivatives (Table 1). Both platforms were φC31-based integrative vectors providing a stable gene expression. pSET152A derivatives were pSAD4 and pSAD3 (already reported in our previous work [38]), where *dbv4* and *dbv3* were placed under the control of the apramycin resistance gene promoter (*aac(3)IVp*). These vectors were previously used to markedly increase the production of GPAs in *Nonomuraea* spp. [38,47]. In pIJ12551, *dbv4* and *dbv3* were placed under the control of the erythromycin resistance gene promotor (*ermEp*). This is the most widely used promoter for gene overexpression in *Streptomyces* spp. [48,49], but it was previously shown to be rather weak in *Nonomuraea* spp. [38]. Complemented strains were named *N. gerenzanensis* Δ*dbv3* pSAD3^+^, *N. gerenzanensis* Δ*dbv4* pSAD4^+^, *N. gerenzanensis* Δ*dbv4* pIJ12551*dbv4*^+^, and *N. gerenzanensis* Δ*dbv3* pIJ12551*dbv3*^+^ (see Table 1).

**Figure 2:**
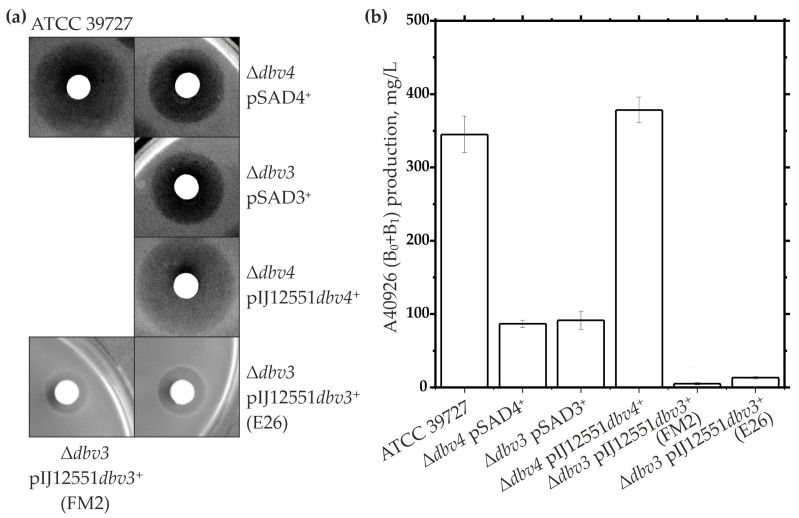
A40926 production is restored in *N. gerenzanensis* Δ*dbv3* and Δ*dbv4* after complementation with native regulatory genes expressed from different platforms. (**a**) *B. subtilis* HB0933 growth inhibition assays in MHA medium; complemented strains demonstrated restoration of antimicrobial activity. Culture samples were collected after 144 h cultivation in FM2 liquid medium (in the case of Δ*dbv3* pIJ12551*dbv3^+^* also in E26) and A40926 was extracted as reported in Materials and Methods. In total, 50 μL of extracts were loaded on 6 mm paper disk. (**b**) Production of A40926 in the complemented strains in comparison to the wild type when cultivated for 144 h in FM2 liquid medium (in case of Δ*dbv3* pIJ12551*dbv3^+^* also in E26). A40926 was extracted and measured by the HPLC as described in Materials and Methods, representing the cumulative concentration of B_0_ and B_1_ congeners. Data represent mean values of three independent experiments ± 2SD.

All the recombinant strains were verified by PCR (Figure S3) and cultivated in FM2 medium for 144 h. Culture extracts at this time point demonstrated restored antimicrobial activity in bioassays (Figure 2a). HPLC analysis of the same extracts further revealed that *N. gerenzanensis* Δ*dbv3* pSAD3^+^ and Δ*dbv4* pSAD4^+^ produced A40926 at approximately 25% of the wild type, while the A40926 production level was completely restored in *N. gerenzanensis* Δ*dbv4* pIJ12551*dbv4^+^,* but it reached only *ca.* 1.5% in *N. gerenzanensis* Δ*dbv3* pIJ12551*dbv3^+^* (Figure 2b). The A40926 production levels of *N. gerenzanensis* Δ*dbv3* pIJ12551*dbv3^+^* were slightly better when monitored in E26 vegetative medium, where they reached 13.25 mg/L (*ca.* 3% of the parental strain productivity in FM2 medium) (Figure 2b).

### 2.2. Cross-Complementation of N. gerenzanensis Δdbv4 and A. teichomyceticus Δtei15* with tei15* and dbv4

As demonstrated in our previous work [30], *dbv4* and tei15* (coding for StrR-like transcriptional regulators) are orthologous, and they have orthologues in all other GPA BGCs. In addition, Dbv4 and its orthologue from *Am. balhimycina* DSM 5908 balhimycin BGC—Bbr [33]—showed similar DNA-binding properties [36]. Regarding this, we speculated that *dbv4* and tei15* might be interchangeable. To investigate such eventual “cross-talk” between PSRs controlling teicoplanin and A40926 production, we “exchanged” the genes coding for StrR-like transcriptional regulators between *N. gerenzanensis* and *A. teichomyceticus*. To achieve this, we introduced either pSET152Atei15* [35] or pIJ12551tei15* plasmids into *N. gerenzanensis* Δ*dbv4*. In parallel, we transferred pSHAD4 (carrying *dbv4*) into the teicoplanin non-producing mutant *A. teichomyceticus* Δtei15* [35]. pSHAD4 was generated by replacing the *aac(3)IV* gene in pSAD4 with the hygromycin resistance gene *hygR*. Such a replacement was necessary since *A. teichomyceticus* Δtei15* is resistant to apramycin. Obtained recombinant strains (validated by PCR as reported in Figure S4) were named *N. gerenzanensis* Δ*dbv4* pSET152Atei15*^+^, *N. gerenzanensis* Δ*dbv4* pIJ12551tei15*^+^, and *A. teichomyceticus* Δtei15* pSHAD4^+^ (Table 1).

**Figure 3:**
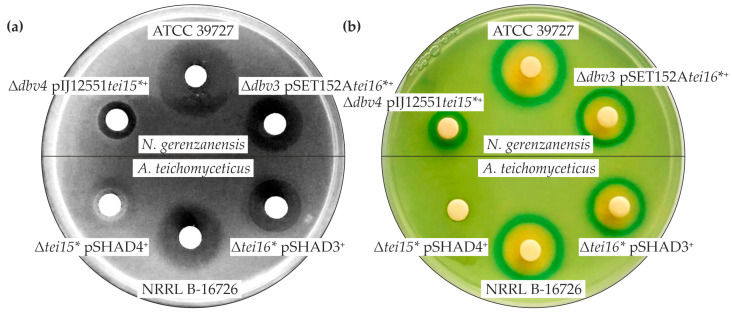
GPA production phenotypes of *N. gerenzanensis* and *A. teichomyceticus* CSRGs mutants complemented with heterologous CSRGs tested by *B. subtilis* HB0933 (**a**) and HB0950 (**b**) growth inhibition assays. *N*. *gerenzanensis* Δ*dbv4* pIJ12551tei15*^+^, Δ*dbv3* pSET152Atei16*^+^, and *A. teichomyceticus* Δtei16* pSHAD3*^+^* inhibited the growth of *B. subtilis* HB0933 and induced X-Gal chromogenic conversion in *B. subtilis* HB0950, indicating restoration of GPA biosynthesis. Samples were collected after 144 h cultivation in FM2 medium in the case of wild type *N. gerenzanensis* and recombinant strains, and after 96 h cultivation in TM1 in the case of *A. teichomyceticus* and its recombinant strains, with the exception of *A. teichomyceticus* Δtei16* pSHAD3^+^ which was grown 96 h in E25 added with 1 g/L l-valine; GPAs were extracted as reported in Materials and Methods. In total, 50 μL of extracts were loaded onto a 6 mm paper disks.

*N. gerenzanensis* recombinant strains were cultivated in FM2 medium, as previously described, and their A40926 production was followed by *B. subtilis* growth inhibition assays and HPLC analyses of the corresponding culture broth extracts. Although no antimicrobial activity against *B. subtilis* HB0933 was detectable for *N. gerenzanensis* Δ*dbv4* pSET152Atei15*^+^ (Figure S5), a small inhibition halo was observed only in the case of *N. gerenzanensis* Δ*dbv4* pIJ12551tei15*^+^ (Figure 3a), but no A40926 was identified through HPLC analyses.

**Figure 4:**
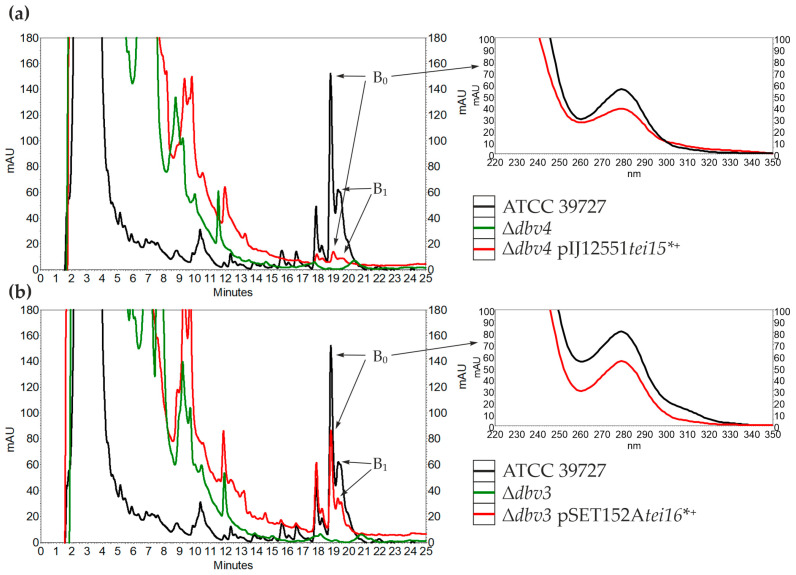
HPLC analysis showing the restoration of A40926 production in *N*. *gerenzanensis* Δ*dbv4* pIJ12551tei15*^+^ (**a**) and Δ*dbv3* pSET152Atei16*^+^ (**b**). *N*. *gerenzanensis* strains were cultivated for 144 h in FM2; A40926 was extracted as described in Materials and Methods and the extracts from Δ*dbv4,* Δ*dbv4* pIJ12551tei15*^+^, Δ*dbv3*, and Δ*dbv3* pSET152Atei16*^+^ were concentrated ten times by lyophilization. Peaks with a typical UV spectrum, corresponding to A40926 B_0_ and B_1_ (indicated by black arrows), were clearly visible in the control *N*. *gerenzanensis* ATCC 39727 (black). In (**a**), A40926 B_0_ and B_1_ were detectable, although at a very low level, in the concentrated extracts from *N*. *gerenzanensis* Δ*dbv4* pIJ12551tei15*^+^(red*)*, but not in those from *N*. *gerenzanensis* Δ*dbv4* (green). In (**b**), A40926 B_0_ and B_1_ were detectable in Δ*dbv3* pSET152Atei16*^+^ concentrated extracts (red) but were undetectable in those from *N*. *gerenzanensis* Δ*dbv3* (green) (for the chromatogram of commercial A40926 standard please refer to Figure S2a).

The poor antimicrobial activity of *N. gerenzanensis* Δ*dbv4* pIJ12551tei15*^+^ was further assessed against *B. subtilis* HB0950 (Figure 3b), a reporter strain containing the *lacZ* gene (coding for β-galactosidase) fused to the *liaI* promoter (*PliaI*), which is able to activate the expression of *lacZ* in response to the cell wall stress caused by lipid II binders [50]. Thus, GPA production could be identified by cultivating *B. subtilis* HB0950 in the presence of X-Gal; as lipid II binders, GPAs induce the chromogenic conversion of X-Gal. Indeed, the chromogenic conversion of X-Gal was induced by extracts obtained from *N. gerenzanensis* ATCC 39727 and *N. gerenzanensis* Δ*dbv4* pIJ12551tei15*^+^, indicating that they both produce A40926 (Figure 3b). Driven by this evidence, the bioactive culture extracts of *N. gerenzanensis* Δ*dbv4* pIJ12551tei15*^+^ were concentrated ten times by lyophilization and A40926 became detectable by HPLC, although at a low concentration (the estimated production was 2 mg/L after 144 h of cultivation in FM2 medium) (Figure 4a). These results indicated that the heterologous expression of tei15* might, albeit at a low efficiency, complement the *dbv4* knockout in *N. gerenzanensis*.

Teicoplanin production was then analyzed cultivating *A. teichomyceticus* Δtei15* pSHAD4^+^ in TM1 medium, previously optimized for teicoplanin production [9], alongside with the parental strain. The culture broth extracts exhibited no antimicrobial activities against either *B. subtilis* HB0933 (Figure 3a) or *B. subtilis* HB0950 (Figure 3b), even after being ten-times concentrated by lyophilization. Confirming the results of growth inhibition assays, no teicoplanin production was detected by means of HPLC. Consequently, *A. teichomyceticus* Δtei15* pSHAD4^+^ was cultivated in a set of different media, testing different combinations of vegetative and production media, previously used for growing other GPA producing strains. These were TM1 [9], ISP2 [51], VSP [11], and the vegetative media E25 [9] modified by adding 1 g/L of l-valine, which is the amino acid precursor contributing to the increased production of teicoplanin [5]. Again, in none of these conditions, *A. teichomyceticus* Δtei15* pSHAD4^+^ exhibited antimicrobial activity and teicoplanin could not be detected by HPLC. Thus, we concluded that *dbv4* was not able to complement the teicoplanin production phenotype in *A. teichomyceticus* Δtei15*.

### 2.3. Cross-Complementation of N. gerenzanensis Δdbv3 and A. teichomyceticus Δtei16* with tei16* and dbv3

In addition to CSRGs coding for StrR-like transcriptional regulators, teicoplanin and A40926 BGCs also carry CSRGs for LuxR-like transcriptional regulators. Differently from StrR-like proteins, Dbv3 and Tei16* are not orthologous [30]; Tei16* has its orthologues coded within the BGCs of other *Actinoplanes-*derived GPAs, and, surprisingly, in feglymycin BGC from *Streptomyces* sp. DSM 11171 [52]. On the other hand, Dbv3 orthologues are coded only in *Nonomuraea-*derived BGCs for dalbaheptides and type V GPAs [47,53]. Thus, we considered it to be particularly intriguing to investigate if a cross-complementation between seemingly unrelated CSRGs might occur.

To achieve this goal, we transferred pSET152Atei16*—previously reported to trigger teicoplanin overproduction in *A. teichomyceticus* [45]—into *N. gerenzanensis* Δ*dbv3* (described above). In parallel, pSHAD3 was transferred into *A. teichomyceticus* Δtei16* teicoplanin non-producing mutant. So-obtained recombinant strains expressing heterologous genes for LuxR-like regulators (validated by PCR as reported in Figure S4) were named *N. gerenzanensis* Δ*dbv3* pSET152Atei16*^+^ and *A. teichomyceticus* Δtei16* pSHAD3^+^ (Table 1).

As illustrated in Figure 3, *N. gerenzanensis* Δ*dbv3* pSET152Atei16*^+^ inhibited the growth of *B. subtilis* HB0933 (Figure 3a) and was able to induce the chromogenic conversion of X-Gal in *B. subtilis* HB0950 (Figure 3b), indicating that tei16* was able to restore A40926 production. HPLC analysis of the concentrated culture extracts of *N. gerenzanensis* Δ*dbv3* pSET152Atei16*^+^ confirmed the complementation of the A40926 production phenotype, with an estimated production reaching 5–6% of the wild type A40926 production levels after 144 h of cultivation in FM2 medium (Figure 4b).

**Figure 5:**
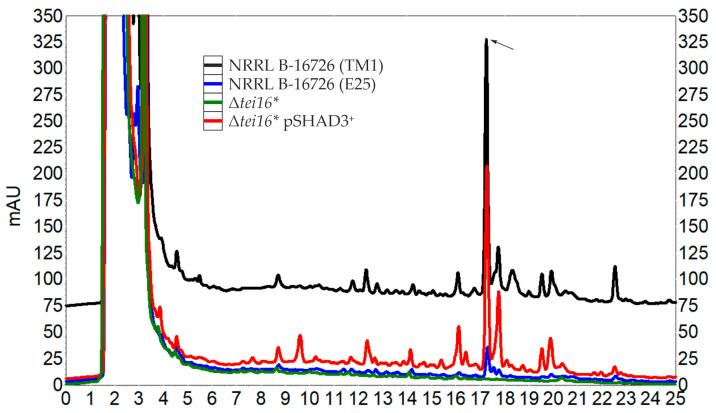
Restoration of teicoplanin production phenotype in *A. teichomyceticus* Δtei16* upon introduction of pSHAD3, as revealed with HPLC. Black and blue chromatograms show the production profile of *A. teichomyceticus* NRRL B-16726 in TM1 and in E25, supplemented with 1g/L of l-valine, after 96 h cultivation, the red chromatogram shows the production profile of *A. teichomyceticus* Δtei16* pSHAD3^+^ in E25 added with 1g/L of l-valine at the same cultivation time point, while the green chromatogram demonstrates the absence of teicoplanin production in *A. teichomyceticus* Δtei16* cultivated in TM1 for 96 h. No production (superimposable profiles not shown) was observed with A. teichomyceticus Δtei16* pSHAD3^+^ in TM1 and with *A. teichomyceticus* Δtei16* in E25 + 1 g/L l-valine. Teicoplanin main peak (factor A_2–2_) is indicated with a black arrow (for the chromatogram of commercial teicoplanin standard, please refer to Figure S2b).

The growth of *A. teichomyceticus* Δtei16* pSHAD3^+^ (carrying *dbv3*) was very poor in TM1 production medium due to severe mycelium fragmentation. Similarly to the case of *A. teichomyceticus* Δtei15* pSHAD4^+^ (see above), we investigated whether some other liquid media might support its better growth and teicoplanin production. Indeed, teicoplanin production was observed when *A. teichomyceticus* Δtei16* pSHAD3^+^ was cultivated in E25 vegetative medium supplemented with 1 g/L of l-valine by means of *B. subtilis* HB0933 (Figure 3a) and HB0950 growth inhibition assays (Figure 3b). The HPLC analysis of culture broth extracts from *A. teichomyceticus* Δtei16* pSHAD3^+^ confirmed the restoration of the teicoplanin production phenotype (Figure 5), showing the production of approximately 150 mg/L of teicoplanin (as the cumulative concentration of five main congeners [9]).

### 2.4. Expression of tei15* and tei16* in the Double dbv3 and dbv4 Knockout Mutant of N. gerenzanensis

Unlike in *A. teichomyceticus* [30,35], the PSRs of A40926 biosynthesis do not act hierarchically; instead, either Dbv4 or Dbv3 control the expression of discrete regulons within *dbv.* Our data demonstrated (see above) that the introduction of either tei15* or tei16* into Δ*dbv4* and Δ*dbv3* mutants of *N. gerenzanensis*, respectively, restored A40926 production to a certain extent. In the case of *N. gerenzanensis* Δ*dbv4* pIJ12551tei15*^+^, such a restoration could be readily explained because both Dbv4 and Tei15* are related StrR-like regulators binding similar operator sequences [35,36]. Hence, Tei15* is probably able to activate only the genes of Dbv4 regulon. The case of *N. gerenzanensis* Δ*dbv3* pSET152Atei16*^+^ is more obscure: since both LuxR-like regulators are distantly related [30], it is possible that the Tei16*-mediated restoration of A40926 production might be unspecific, activating different genes from Dbv4 and Dbv3 regulons, or even involving some other players.

To evaluate if the introduction of tei15* and tei16* specifically restores A40926 production in Δ*dbv4* and Δ*dbv3* mutants, we decided to create a double *dbv3* and *dbv4* knockout mutant of *N. gerenzanensis*. Thus, both genes together were replaced with the *oriT-aadA* cassette using the λ-Red-mediated recombineering approach [43], as described in Materials and Methods (Figure S1). The obtained mutant was named *N. gerenzanensis* Δ*dbv3–4* and was verified by PCR (Figure S1). As expected, *N. gerenzanensis* Δ*dbv3–4* was unable to produce A40926 (Figure S6a,b). We further introduced either tei15* or tei16* in the double knockout mutant; obtained strains were called *N. gerenzanensis* Δ*dbv3–4* pIJ12551tei15*^+^ and *N. gerenzanensis* Δ*dbv3–4* pSET152Atei16*^+^, respectively (Table 1), and were verified by PCR (Figure S7). In both recombinants, we observed no restoration of A40926 production or the induction of any other antimicrobial activities (Figure S6a). Considering these results, we could conclude that either the tei15*- or tei16*-mediated restoration of A40926 production in *N. gerenzanensis* Δ*dbv4* and Δ*dbv3*, respectively, is rather due to the specific activation of Dbv4 and Dbv3 regulons.

### 2.5. Cross-Overexpression of dbv and tei CSRGs in N. gerenzanensis ATCC 39727 and A. teichomyceticus NRRL B-16726

**Figure 6:**
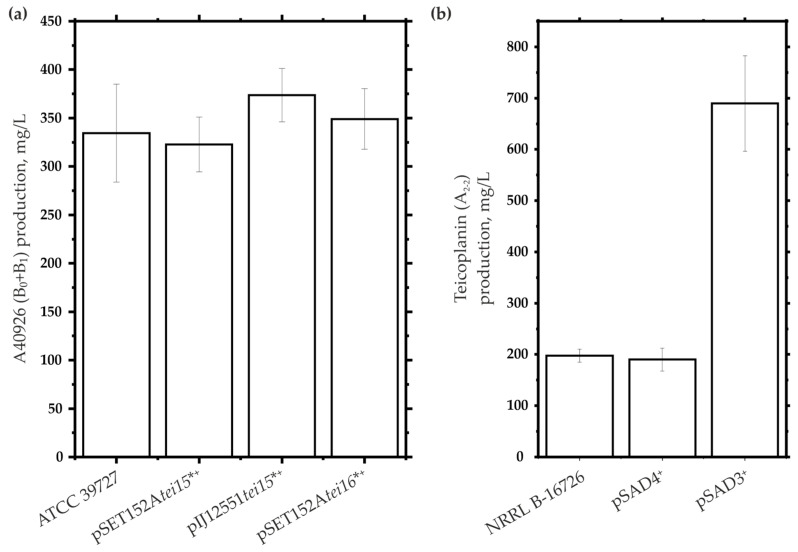
GPA production in *N. gerenzanensis* (**a**) and *A.* t*eichomyceticus* (**b**) strains, expressing heterologous CSRGs. (**a**) A40926 production levels in FM2 medium by *N. gerenzanensis* ATCC 39727, *N. gerenzanensis* pSET152tei15*^+^, *N. gerenzanensis* pIJ12551tei15*^+^, and *N. gerenzanensis* pSET152tei16*^+^ after 144 h growth; A40926 was extracted and measured as described in Materials and Methods. (**b**) Teicoplanin production in TM1 medium by *A. teichomyceticus* NRRL B-16726, *A. teichomyceticus* pSAD4^+^, and *A. teichomyceticus* pSAD3^+^ after 96 h growth. Teicoplanin was extracted and measured as described in Materials and Methods. Data represent mean values of three independent experiments ± 2SD.

Since tei15* and tei16* are able to restore A40926 production in *N. gerenzanensis* Δ*dbv3* and Δ*dbv4*, respectively, we tested if their overexpression in *N. gerenzanensis* ATCC 39727 would affect antibiotic production. To this aim, we created *N. gerenzanensis* pSET152Atei15*^+^, pIJ12551tei15*^+^, and pSET152Atei16*^+^ recombinant strains (verified by PCR, see Figure S4), but their A40926 production levels in FM2 medium did not exceed that of the wild type (Figure 6a).

We reproduced this approach also for *A. teichomyceticus* NRRL B-16726, generating *A. teichomyceticus* pSAD4^+^ and *A. teichomyceticus* pSAD3^+^ recombinants overexpressing the heterologous CSRGs *dbv 4* and *dbv3,* respectively. The obtained recombinants were cultivated in TM1 medium and the teicoplanin production was measured by HPLC. We found that *A. teichomyceticus* pSAD4^+^ produced teicoplanin at the levels of the wild type, whereas the teicoplanin production was significantly increased in *A. teichomyceticus* pSAD3^+^, reaching *ca.* 700 mg/L (Figure 6b).

## 3. Discussion

Previous works reported that StrR-like pathway-specific regulators coded in different GPA BGCs were able to “cross-talk” in vivo between different pathways. For instance, StrR-like pathway-specific regulators Bbr and Ajr (the first from the balhimycin BGC in *Am. balhimycina* DSM 5908 and the second from ristocetin BGC in *Am. japonicum* MG417-CF17) could replace each other in governing the reciprocal GPA synthesis [12]. Both Bbr and Ajr share more than an 80% amino acid sequence identity, reflecting the similarity of corresponding BGCs deriving from *Amycolatopsis* spp. Similarly, the heterologous expression of Dbv4 in *Nonomuraea coxensis* DSM 45129 was able to increase GPA production levels in the latter [47]. Additional evidence also exists showing that StaQ—a PSR controlling A47934 [54] biosynthesis in *Streptomyces toyocaensis* NRRL 15009 [55]—was able to upregulate the expression of heterologous GPA BGCs [56].

In the current study, we investigated whether *dbv4* and tei15* (sharing an only 53% amino acid sequence identity) would be able to “cross-talk” between rather diverged GPA biosynthetic pathways deriving from species belonging to different families, *Micromonosporaceae* in the case of teicoplanin and *Streptosporangiaceae* in the case of A40926. Our results did not show a clear reciprocal behavior of the two StrR-like pathway-specific regulators. In particular, the overexpression of tei15* was able to restore A40926 production in *N. gerenzanensis* Δ*dbv4*, but to a very low extent, restoring only *ca.* 1% of A40926 production in comparison to the wild type. Although the activating effect of tei15* was slight, it was rather specific, since the overexpression of tei15* was not able to restore A40926 production in *N. gerenzanensis* Δ*dbv3–4.* At the same time, *dbv4* was not able to restore teicoplanin production in *A. teichomyceticus* Δtei15* at all.

The obtained results portray that Dbv4 and Tei15* are less functionally related than was previously believed, despite their clear orthology. Although they share the basic architecture of structural domains, as well as a high degree of amino acid sequence similarity, their DNA-binding activity is likely shaped in a way that they could not “cross-talk”. These results corroborate the previous findings of Tei15* DNA-binding activities in vitro [17], shown to be slightly different from the DNA-binding activities of Bbr [13]. In turn, other work demonstrated that Bbr and Dbv4 had rather similar DNA-binding properties [19]. Anyhow, further investigations are needed on their mode of action at the transcriptional level. A high-resolution phylogenetic analysis of all StrR-like proteins deriving from the *Actinobacteria* phylum might also be useful to better understand their evolutionary and reciprocal relations.

The emerging picture with LuxR-like pathway-specific regulators appeared to be even more puzzling. Dbv3 and Tei16* are not orthologous, sharing only 30% of an amino acid sequence identity and possessing dramatically different regulons [35,36,37]. Surprisingly, our results revealed that *dbv3* is able to fully restore the teicoplanin production phenotype in *A. teichomyceticus* Δtei16*, while *N. gerenzanensis* Δ*dbv3* pSET152tei16*^+^ accumulated only hardly traceable amounts of A40926. We also achieved a significant increase in teicoplanin production when *dbv3* was overexpressed in wild type *A. teichomyceticus*. Peculiarly, the overexpression of tei16* was not able to restore A40926 production in *N. gerenzanensis* Δ*dbv3–4*, implying that Tei16* specifically activates only the regulon of Dbv3, which is not enough to achieve A40926 production in the absence of *dbv4.*

To conclude, if tei15* and *dbv4* were only partially able (tei15*) or unable (*dbv4*) to “cross-talk” between the pathways, *dbv3* and tei16* appeared more active in influencing heterologous pathways. In particular, *dbv3* exerted a significant effect on teicoplanin production in *A. teichomyceticus* NRRL B-16726. Although the molecular background of these events is yet to be elucidated and merits further investigations, the results achieved in this work contribute to a throughout understanding of GPAs biosynthesis regulation, providing additional clues to comprehend their evolution. In addition, they might offer novel molecular tools for engineering producing actinomycetes and improving their GPA production, rendering the manufacturing processes for these antibiotics more sustainable and reducing their production costs.

## 4. Materials and Methods

### 4.1. Bacterial Strains and Growth Conditions 

All strains and plasmids used in this work are listed in Table 1. *N. gerenzanensis* ATCC 39727, *A. teichomyceticus* NRRL B-16726 (ATCC 31121), and the derived recombinant strains were routinely cultivated on ISP3 agar medium at 30 °C. For A40926 production, *N. gerenzanensis* and recombinants were fermented in 50 mL of E26 vegetative medium for 72 h, and then 10% (*v/v*) of the preculture was inoculated in 100 mL of FM2 production medium [11]. *A. teichomyceticus* and its derived recombinants were fermented in 50 mL of E25 vegetative medium for 72 h, and then 10% (*v/v*) of the preculture was inoculated in 100 mL of TM1 production medium for teicoplanin production [9]. All the strains were grown on an orbital shaker in baffled Erlenmeyer flasks at 30 °C, 220 rpm. *Escherichia coli* DH5α was used for routine DNA cloning. *E. coli* ET12567 pUZ8002^+^ [48] was used as a donor for intergeneric mattings with *Nonomuraea* and *Actinoplanes. E. coli* strains carrying recombinant plasmids were grown in Lysogeny broth (LB) agar at 37 °C supplemented with 100 μg/mL of apramycin–sulfate, 100 μg/mL of spectinomycin, 50 μg/mL of hygromycin B, 50 μg/mL of kanamycin–sulfate, and 25 μg/mL of chloramphenicol when necessary. 

**Table 1:** Bacterial strains and plasmids used in this work.

Plasmids	Description	Source or Reference
pIJ778	Template vector for the amplification of *oriT-aadA* cassette for PCR-targeted mutagenesis	[57]
A40Y	SuperCos1 derivative, including 22 kb fragment of *dbv* cluster (*dbv1*–*dbv17*)	[58]
A40dbv3::aadA	A40Y derivative with *dbv3* replaced by *oriT-aadA* cassette	This work
A40dbv4::aadA	A40Y derivative with *dbv4* replaced with *oriT-aadA*	This work
A40dbv3–4::aadA	A40Y derivative with *dbv3* and *dbv4* replaced with *oriT-aadA* cassette derived from plasmid pIJ778	This work
pKC1132	Suicide plasmid for gene knockouts, Am^R^	[59]
pKCKOD3	pKC1132 derivative carrying *dbv3* flanking regions surrounding *oriT-aadA*, Am^R^	This work
pKCKOD4	pKC1132 derivative carrying *dbv4* flanking regions surrounding *oriT-aadA*, Am^R^	This work
pKCKOD3–4	pKC1132 derivative carrying *dbv3–4* flanking regions surrounding *oriT-aadA*, Am^R^	This work
pSAD3	pSET152A derivative carrying *dbv3* under the control of *aac(3)IVp*, Am^R^	[38]
pSAD4	pSET152A derivative carrying *dbv4* under the control of *aac(3)IVp*, Am^R^	[38]
pIJ10700	Template vector for the amplification of *hygR*	[43]
pSHAD3	pSAD3 derivative, Hg^R^	This work
pSHAD4	pSAD4 derivative, Hg^R^	This work
pIJ12551	ϕC31-actinophage-based integrative vector for gene expression under the control of ermE* promoter, Am^R^	[46]
pIJ12551*dbv4*	pIJ12551 derivative carrying *dbv4* under the control of ermE*, Am^R^	This work
pIJ12551*dbv3*	pIJ12551 derivative carrying *dbv3* under the control of ermE*, Am^R^	This work
pSET152Atei15*	pSET152A derivative carrying tei15* under the control of *aac(3)IVp*, Am^R^	[35]
pSET152Atei16*	pSET152A derivative carrying tei16* under the control of *aac(3)IVp*, Am^R^	[35]
pIJ12551tei15*	pIJ12551 derivative carrying tei15* under the control of ermE*	This work
**Bacterial Strains**		
*N. gerenzanensis*	Wild type, A40926 producer	ATCC 39727
*N. gerenzanensis* Δ*dbv3*	Wild type derivative with *dbv3* replaced by *oriT-aadA* cassette	This work
*N. gerenzanensis* Δ*dbv4*	Wild type derivative with *dbv4* replaced by *oriT-aadA* cassette	This work
*N. gerenzanensis Δdbv3–4*	Wild type derivative with *dbv3–4* replaced by *oriT-aadA* cassette	This work
*N. gerenzanensis* Δ*dbv3* pSAD3^+^	*N. gerenzanensis* Δ*dbv3* derivative carrying pSAD3	This work
*N. gerenzanensis* Δ*dbv4* pSAD4^+^	*N. gerenzanensis* Δ*dbv4* derivative carrying pSAD4	This work
*N. gerenzanensis* Δ*dbv4* pIJ12551*dbv4*^+^	*N. gerenzanensis* Δ*dbv4* derivative carrying pIJ12551*dbv4*	This work
*N. gerenzanensis* pSET152Atei15*^+^	Wild type derivative carrying pSET152Atei15*	This work
*N. gerenzanensis* pSET152Atei16*^+^	Wild type derivative carrying pSET152Atei16*	This work
*N. gerenzanensis* pIJ12551tei15*^+^	Wild type derivative carrying pIJ12551tei15*	This work
*N. gerenzanensis* Δ*dbv4* pSET152Atei15*^+^	*N. gerenzanensis* Δ*dbv4* derivative carrying pSET152Atei15*	This work
*N. gerenzanensis* Δ*dbv3* pSET152Atei16*^+^	*N. gerenzanensis* Δ*dbv3* derivative carrying pSET152Atei16*	This work
*N. gerenzanensis* Δ*dbv4* pIJ12551*dbv4*^+^	*N. gerenzanensis* Δ*dbv4* derivative carrying pIJ12551*dbv4*	This work
*N. gerenzanensis* Δ*dbv3–4* pIJ12551tei15*^+^	*N. gerenzanensis* Δ*dbv3–4* derivative carrying pIJ12551tei15*	This work
*N. gerenzanensis* Δ*dbv3–4* pSET152Atei16*^+^	*N. gerenzanensis* Δ*dbv3–4* derivative carrying pSET152Atei16*	This work
*A. teichomyceticus*	Wild type, teicoplanin producer	NRRL-B16726
*A. teichomyceticus* pSAD3^+^	Wild type derivative carrying pSAD3	This work
*A. teichomyceticus* pSAD4^+^	Wild type derivative carrying pSAD4	This work
*A. teichomyceticus* Δtei15*	Wild type derivative with tei15* gene replaced by *oriT-aac(3)IV* cassette	[35]
*A. teichomyceticus* Δtei16*	Wild type derivative with tei16* gene replaced with *oriT-aac(3)IV* cassette	[35]
*A. teichomyceticus* Δtei15* pSHAD4^+^	*A. teichomyceticus* Δtei15* derivative carrying pSHAD4	This work
*A. teichomyceticus* Δtei16* pSHAD3^+^	*A. teichomyceticus* Δtei16* derivative carrying pSHAD3	This work
*E. coli* DH5α	General cloning host	MBI Fermentas, US
*E. coli* ET12567 pUZ8002^+^	(*dam-13*::Tn*9 dcm-6*), pUZ8002^+^ (Δ*oriT*), used for conjugative transfer of DNA	[60]
*E. coli* BW25113 pIJ790^+^	λ-Red-mediated recombineering host	[43]
*B. subtilis* HB0933	CU1065 *liaR*::*kan*	[61]
*B. subtilis* HB0950	CU1065 SPβ2Δ2::Tn*917*::Φ(*PliaI*-74-*cat*-*lacZ*)	[50]

### 4.2. Extraction of Genomic DNA

Extraction of genomic DNA from *N. gerenzanensis* and *A. teichomyceticus* strains was conducted following the Kirby procedure [48]. Prior to DNA isolation, *N. gerenzanensis* and *A. teichomyceticus* strains were cultivated in 250 mL baffled Erlenmeyer flasks containing 50 mL of E25 or E26 on an orbital shaker at 220 rpm and 30 °C for 96 h. 

### 4.3. Inactivation of dbv3 and dbv4 in N. gerenzanensis

First of all, *dbv3, dbv4,* and both *dbv3* and *dbv4* genes together were replaced on A40Y cosmid (containing the majority of *dbv* BGC genes) [58] with a spectinomycin/streptomycin resistance cassette (*oriT-aadA*, derived from pIJ778 [57]) using the λ-Red-mediated recombineering approach [43]. The obtained A40Y derivatives were named A40dbv4::aadA, A40dbv3::aadA, and A40dbv3–4::aadA, respectively. The primer pairs dbv3_P1/dbv3_P2, dbv4_P1/dbv4_P2, and dbv3_P1/dbv4_P2 were used for the replacement and are listed in Table 2. Then, using generated recombinant cosmids as templates, we amplified *aadA–oriT* with *ca*. 2 Kbp flanking regions of *dbv3*, *dbv4,* and *dbv3–4* (which were previously replaced by *oriT-aadA* cassette) and cloned the amplicons into pKC1132 [59] suicide vector (Table 1). The latter carries the apramycin resistance gene *aac(3)IV* and neither phage integration systems genes nor replicon regions, depending on the homologous recombination for the integration into the chromosome of *N. gerenzanensis*. Amplicons were obtained with dbv3KO_F/R, dbv4KO_F/R, and dbv3KO_F/dbv4KO_R using Q5 High-Fidelity DNA Polymerase (New England Biolabs, Ipswich, MA, United States), digested with *Hind*III/*Spe*I restriction endonucleases and cloned into pKC1132 via *Hind*III/*Xba*I recognition sites. In this way, pKCKOD3, pKCKOD4, and pKCKOD3–4 knockout suicide plasmids were generated (Table 1) and introduced into *N. gerenzanensis* ATCC 39727 via an intergeneric conjugation with *E. coli* ET12567 pUZ8002^+^. The obtained transconjugants were spectinomycin (Sp^R^) and apramycin resistant (Am^R^), indicating that pKCKOD3, pKCKOD4, or pKCKOD3–4 successfully integrated into the chromosome via a single crossing over event. pKCKOD3^+^, pKCKOD4^+^, and pKCKOD3–4^+^ were then cultivated several times on ISP3 solid medium without any selective antibiotics. After the last cultivation passage, each strain was reseeded on a fresh ISP3 plate with no antibiotics using an exhaustive streak to obtain single colonies. Finally, these single colonies were analyzed by replica-plating to identify Am^S^, Sp^R^ ones, where the second crossing-over event took place, replacing *dbv3, dbv4,* or *dbv3–4* genes with *aadA-oriT.* The obtained mutants were named Δ*dbv3,* Δ*dbv4,* and Δ*dbv3–4* and were verified by PCR, using the 3/4KOVER_F/R primer pair (Table 2). A detailed scheme displaying all steps of our knockout approach is provided in ESM Figure S1.

### 4.4. Generation of the Recombinant Plasmids

First, tei15*, dbv4, and *dbv3* pathway-specific regulatory genes were cloned into the pIJ12551 plasmid [46] under the control of the ermE* promotor. To achieve this, coding sequences of tei15*, *dbv4,* and *dbv3* were amplified from the genomic DNA of *A. teichomyceticus* and *N. gerenzanensis*, respectively, and cloned into pIJ12551 plasmid via *Nde*I/*NotI* recognition sites. Amplicons were generated using Q5 High-Fidelity DNA Polymerase (New England Biolabs, Ipswich, MA, United States), and the oligonucleotide primers are listed in Table 2. Generated plasmids were named pIJ12551tei15*, pIJ12551*dbv4*, and pIJ12551*dbv3* (Table 1), verified by restriction mapping and sequencing.

We also generated pSAD3 and pSAD4 [21] derivatives carrying the hygromycin resistance gene (*hygR*, originating from pIJ10700 [43]) instead of *aac(3)IV,* obtaining pSHAD3 and pSHAD4 (Table 1). This was achieved by digesting the pSAD3 and pSAD4 with *Xho*I restriction endonuclease, followed by ends blunting using Shrimp Alkaline Phosphatase (New England Biolabs, Ipswich, MA, United States). So-prepared backbones were ligated with *hygR* amplicon, obtained from pIJ10700 using Q5 High-Fidelity DNA Polymerase and hygR_R/F primer pair. The obtained plasmids were verified by restriction mapping and sequencing.

### 4.5. Conjugative Transfer of Plasmids into N. gerenzanensis and A. teichomyceticus

The conjugative transfer of plasmids (see Table 1 for the complete list, including those already available from other works and those generated in this work) into *N. gerenzanensis* and *A. teichomyceticus* was performed essentially as described previously [38,62]. All recombinant plasmids were transferred individually into the non-methylating *E. coli* ET12567 pUZ8002^+^, and the resulting derivatives were used as donor strains for intergeneric conjugation. To verify the integration of plasmids, target genes or *aac(3)IV* were amplified by PCR from the genomic DNA isolated from the recombinant strains. 

To prepare the fresh vegetative mycelium of *N. gerenzanensis* prior to a conjugal transfer, one vial of WCB was inoculated into 50 mL of VSP medium (250 mL of Erlenmeyer flask with 10 glass beads of ø5 mm) and incubated for 48 h on the orbital shaker at 30 °C, 220 rpm. The mycelium was collected by centrifugation (10 min, 3220× *g*), washed twice with sterile 20% *v/v* glycerol, resuspended in the same solution to a final volume of 20 mL, and stored at −80 °C. In total, 1 mL of mycelial suspension was mixed with approximately 10^9^ of donor *E. coli* cells, and the mixtures were plated on well dried VM0.1 agar plates supplemented with 20 mM of MgCl_2_. After 12–16 h of incubation at 30 °C, each plate was overlaid with 1 mL of sterile deionized water containing 1.25 mg of apramycin–sulfate and 750 μg of nalidixic acid sodium salt. Transconjugants were selected as they are resistant to 50 μg/mL of apramycin–sulfate.

Spore suspensions of *A. teichomyceticus* were prepared from lawns grown on ISP3 agar for 7 days at 30 °C. Sporangia from one plate were collected in deionized water and filtered through one layer of Miracloth (Merck KGaA, Darmstadt, Germany) to remove vegetative mycelial fragments. Then, sporangia were incubated in an orbital shaker at 30 °C until spores were released from sporangia. Then, spores were centrifuged (3220× *g* for 15 min) and resuspended in 1 mL of 15% *v/v* glycerol, and stored at −80 °C. For conjugation, approx. 10^6^ spores were mixed with 10^7^ *E. coli* donor cells and plated on SFM agar plates supplemented with 20 mM of MgCl_2_. The overlay for the selection of transconjugants was performed as described previously for *N. gerenzanensis*.

### 4.6. Microbial Growth Inhibition Assays

To prepare the plates for bioassays, *Bacillus subtilis* HB0933 [61] and HB0950 [50] were grown in LB liquid medium at 37 °C in a shaker (150 rpm) for 15–16 h. Then, 10% (*v/v*) of the overnight culture was inoculated in fresh LB medium and left to grow under the same incubation conditions up to 0.6 OD_600nm_. Subsequently, 100 µL of so-obtained *B. subtilis* culture were inoculated into 30 mL of Muller Hinton Agar (MHA). In the case of *B. subtilis* HB0950, an additional 50 µg/mL of 5-bromo-4-chloro-3-indolyl-β-d-galactoside (X-Gal) were added to the agar to observe the GPA-induced chromogenic conversion. Then, 6 mm paper disks socked with antibiotic extracts or agar plugs were placed on the surface of these plates. The plates were incubated overnight at 37 °C, and growth inhibition halos and X-Gal chromogenic conversion were monitored. 

### 4.7. HPLC Analysis of Teicoplanin and A40926

GPAs were extracted by mixing 1 volume of broth with 1 volume of borate buffer [100 mM H_3_BO_3_ (Sigma-Aldrich, St. Louis, MO, USA), 100 mM NaOH (Sigma-Aldrich), pH 12]. The extraction of teicoplanin was performed by shaking samples on a rotary shaker at 200 rpm and 37 °C for 45 min, then the samples were centrifuged (16,000× *g* for 15 min) to obtain debris-free supernatants according to the protocol reported in [9]. Extracts containing A40926 were centrifuged (16,000× *g* for 15 min), and the supernatants were incubated at 50 °C for 1 h, following the procedure reported in [58]. 

When lyophilization was needed to concentrate the samples containing low amounts of GPAs, the supernatants were lyophilized in a VirTis Sentry vacuum chamber for 24 h and subsequently resuspended in a decreased volume of distilled water in order to concentrate the sample ten times.

HPLC was performed using a VWR Hitachi diode array L-2455 HPLC system with detection at 254 nm for A40926 and 236 nm for teicoplanin. Samples were estimated by injecting 50 μL of sample onto a 5 μm particle size Ultrasphere ODS (Beckman, Brea, California, Stati Uniti) HPLC column (4.6 by 250 mm) or Hypersil GOLD (Thermo Fisher Scientific, Waltham, MA, USA) HPLC column (4.6 by 250 mm). A40926 and teicoplanin samples were eluted at a flow rate of 1 mL/min with a 30 min linear gradient from 15 to 64% of phase B. Phase A was 32 mM HCOONH_4_ (pH 7)—CH_3_CN [90:10 (*v/v*)], and phase B was 32 mM HCOONH_4_ (pH 7)—CH_3_CN [30:70 (*v/v*)]. A volume of 50 μL of a pure sample of 150 μg/mL of A40926 (Sigma-Aldrich, St. Louis, MO, United States) and 100 μg/mL of teicoplanin (Sigma-Aldrich, St. Louis, MO, USA) were used as the standards.

## Figures and Tables

**Table 2:** Oligonucleotide primers used in this work.

Name	Nucleotide Sequence (5′-3′)	Purpose
dbv3KO_R	TTTACTAGTCCCCGATGAGCCTCGGTCC	Amplification of *dbv3* with 2 Kbp flanks
dbv3KO_F	TTTAAGCTTGCCGATCAGACCGGTGCCG	
dbv4KO_R	CCTCGCGAGCTCGTAGCCG	Amplification of *dbv4* with 2 Kbp flanks
dbv4KO_F	CGTGGAAGCGCAGTGCCTC	
3/4KOVER_R	GATATCCTGCCCGAGGCCG	Verification of *dbv3*, *dbv4*, and *dbv3–4* knockouts
3/4KOVER_F	CCAGATGCTGCAGGCGCGA	
dbv3_P1	GCAAACCAAGTCGACGAACCGCTTGGGGGACGAGCAAGAATTCCGGGGATCCGTCGACC	Replacement of *dbv3* with *oriT-aadA* within A40Y
dbv3_P2	GCCCGAGGCCGGCGAATTCGGCTTGTCGAACTCTTCGCTTGTAGGCTGGAGCTGCTTC	
dbv4_P1	CCCCGGCTCCGATATGACGCTAATCGAATCGGAGGCTAGATTCCGGGGATCCGTCGACC	Replacement of *dbv4* with *oriT-aadA* within A40Y
dbv4_P2	GTCGCTCTACATACGGCCGCCCGGCTCATCCACTCGTGCTGTAGGCTGGAGCTGCTTC	
dbv3_FWpIJ	AAAACATATGCTGTTCGGGCGAGATCGT	Cloning of *dbv3* into pIJ12551
dbv3_RVpIJ	AAAAGCGGCCGCCTACAGCCGCACTGCCTC	
dbv4_FWpIJ	AAAAAACATATGGACCCGACGGGAGTTGACATA	Cloning of *dbv4* into pIJ12551
dbv4_RVpIJ	TTTATTAGCGGCCGCTCATCCAGCGGCCAGATCGGTCG	
tei15_FWpIJ	GGGCATATGACACCTGACGAAGAG	Cloning of tei15* into pIJ12551
tei15_RVpIJ	AAAAGCGGCCGCTCAGCTCGCCATC	
pSET_ver_F	GCATCGGCCGCGCTCCCGA	Verification of tei15* and tei16* cloned into pSET152A
tei15*_ver_R	CAGCTCAGCGCCGCTGAGCA	Verification of tei15* cloned into pSET152Atei15*
tei16*_ver_R	CTCGCACACGCCCGGGCC	Verification of tei16* cloned into pSET152Atei16*
hygR_F	GATACACCAAGGAAAGTCT	Amplification of *hygR*
hygR_R	TGTAGGCTGGAGCTGCTTC	
aac(3)IV_Fw	ACCGACTGGACCTTCCTTCT	Verification of apramycin resistance cassette
aac(3)IV_Rv	TCGGTCAGCTTCTCAACCTT	

## Data Availability

All data, strains, and plasmids are available from the corresponding author upon reasonable request.

## Supplementary Materials

The following supporting information can be downloaded at: https://www.mdpi.com/article/10.3390/antibiotics12040641/s1, Figure S1: A scheme depicting the generation of *dbv3*, *dbv4*, and *dbv3–4* knockout mutants of *N. gerenzanensis*; Figure S2: Chromatograms of the commercial standards of A40926 and teicoplanin; Figure S3: Photographs demonstrating PCR verification of genotypes of the complemented *N. gerenzanensis* mutants generated throughout this study; Figure S4: Photographs demonstrating PCR verification of genotypes of cross-complemented mutants of *N. gerenzanensis* and *A. teichomyceticus* generated throughout this study; Figure S5: A40926 production in *N. gerenzanensis* Δ*dbv4* pSET152A*tei15*^+^* is not restored as seen from *B. subtilis* HB0933 growth inhibition assay (MH medium); Figure S6: *N. gerenzanensis* Δ*dbv3–4* is unable to produce A40926 as demonstrated by *B. subtilis* HB0933 growth inhibition assay (MH medium) and HPLC analysis; Figure S7: Photographs demonstrating PCR verification of genotypes of *N. gerenzanensis* and *A. teichomyceticus* strains overexpressing heterologous CSRGs generated throughout this study.
