# Supplementary material for: Cross-Talking of Pathway-Specific Regulators in Glycopeptide Antibiotics (Teicoplanin and A40926) Production
Source: Antibiotics (Basel). 2023 Mar 24;12(4):641. doi: 10.3390/antibiotics12040641 (PMC10135024; doi:10.3390/antibiotics12040641)
Supplement: Supplementary file 1 [file antibiotics-12-00641-s001.zip › antibiotics-2290833-supplementary.pdf]

Electronic supplementary materials

Cross-Talking of Pathway-Specific Regulators in Glycopeptide Antibiotics (Teicoplanin and A40926) Production

Andrés Andreo-Vidal [1], Oleksandr Yushchuk [1,2,*], Flavia Marinelli [1] and Elisa Binda [1]

[1] Department of Biotechnology and Life Sciences, University of Insubria, via J. H. Dunant 3, 21100 Varese, Italy
[2] Department of Genetics and Biotechnology, Ivan Franko National University of Lviv, 79005 Lviv, Ukraine
* Correspondence: oleksandr.yushchuk@uninsubria.it

Supplementary figures

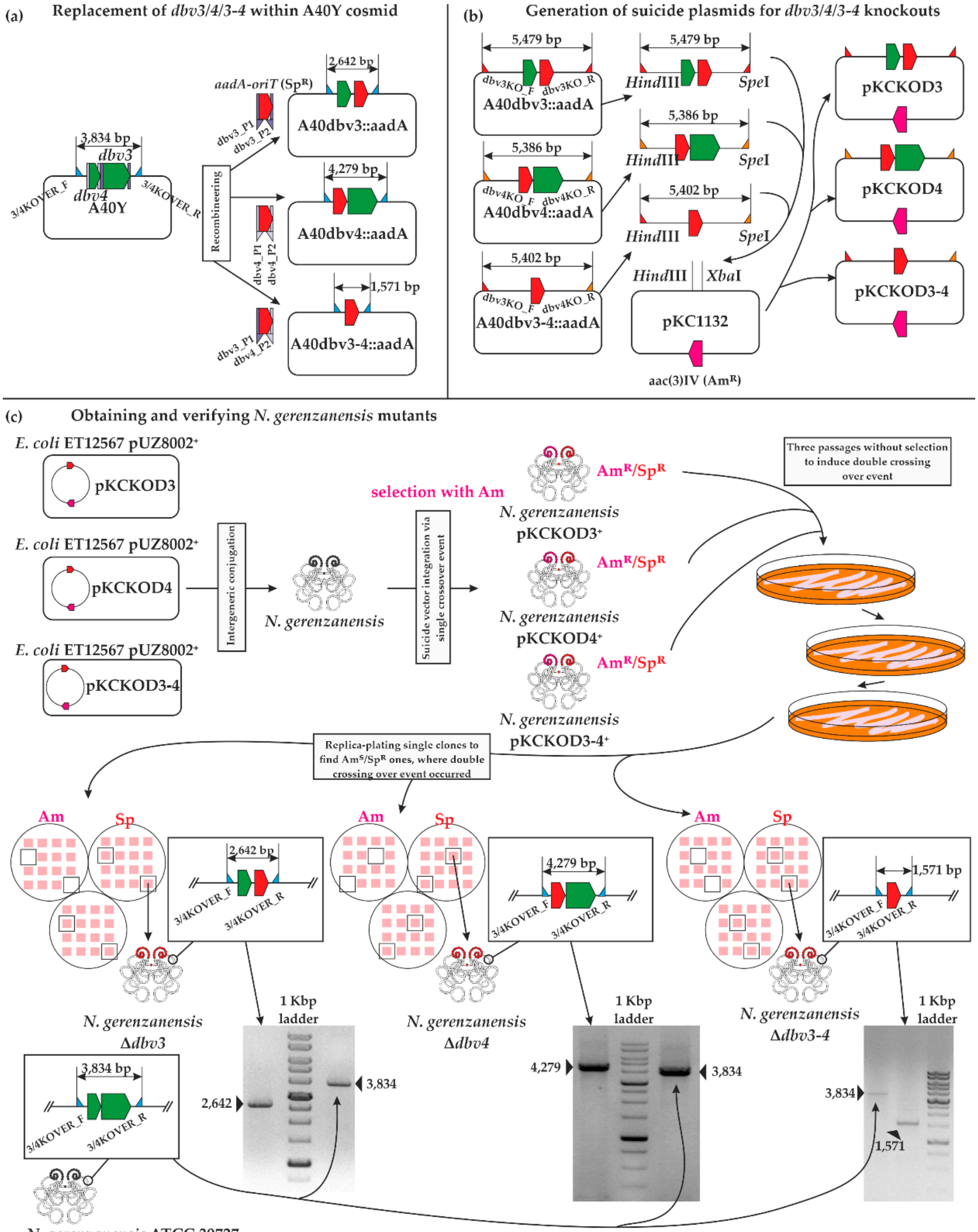

Figure S1. A scheme depicting the generation of *dbv3*, *dbv4*, and *dbv3-4* knockout mutants of *N. gerenzanensis*. a) Replacement of *dbv3*, *dbv4* and both genes together within A40Y cosmid, yielding A40dbv3::aadA, A40dbv4::aadA and A40dbv3-4::aadA, respectively. *dbv3* and *dbv4* are represented as green arrows and *oriT-aadA* as red arrow. Primers to replace *dbv3* are shown in purple triangles while primers to replace *dbv4* are shown as grey triangles. 3/4KOVER primers, which are illustrated as blue triangles, were used to verify the mutants. b) *oriT-aadA* with *ca.* 2 Kbp flanking regions were amplified using dbv3KO_F/R (red triangles), dbv4KO_F/R (orange triangles) or dbv3KO_F/dbv4KO_R, and introduced in pKC1132 suicide vector. Resulting plasmids were named pKCKOD3, pKCKOD4, and pKCKOD3-4. c) Obtainment and verification of *N. gerenzanensis* Δ*dbv3*, Δ*dbv4* and Δ*dbv3-4* strains. Mentioned oligonucleotide primers are given in Table 2, main text.

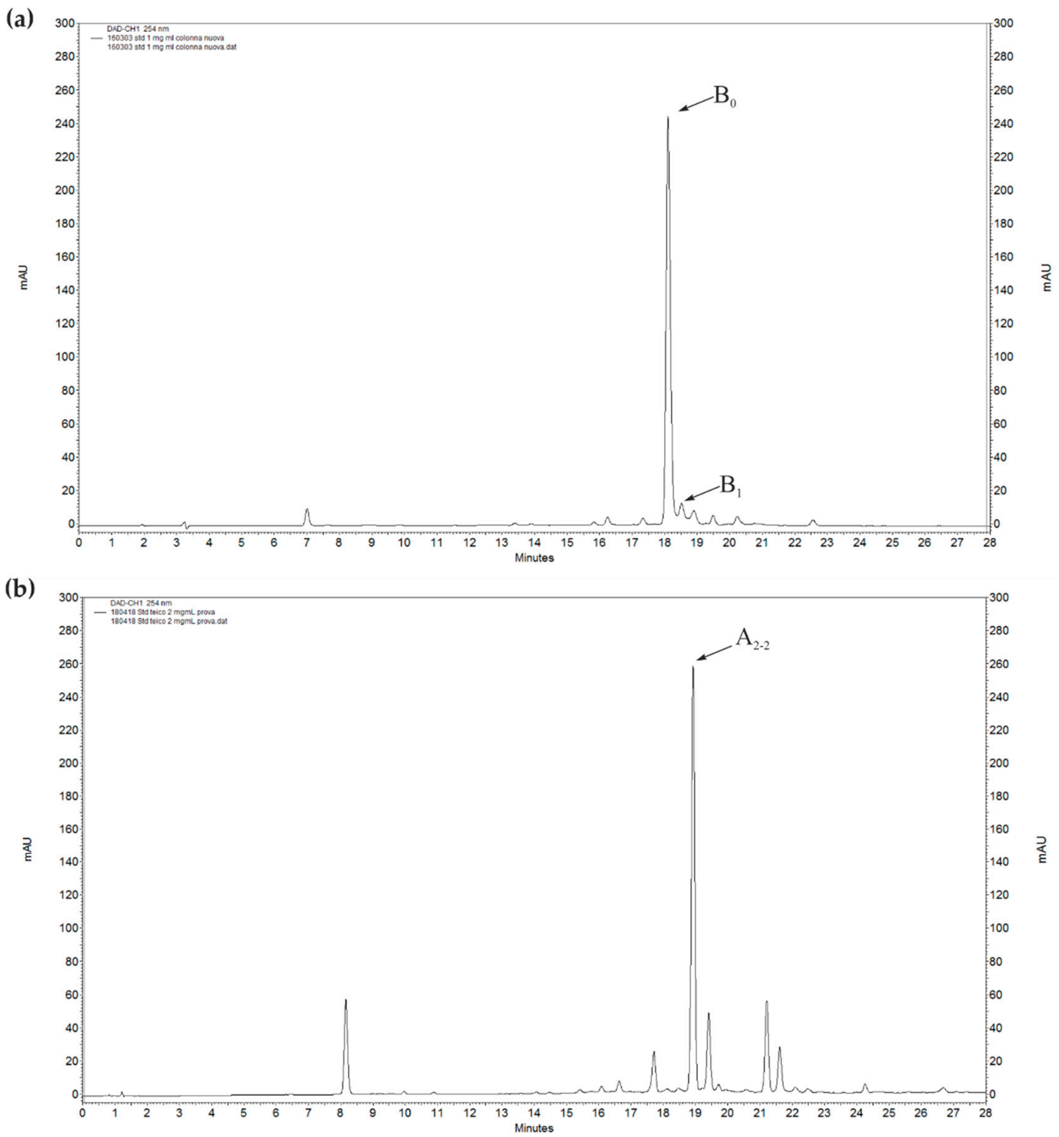

Figure S2. Chromatograms obtained for commercial standards of A40926 (a) and teicoplanin (b) under HPLC conditions described in Materials and Methods. A40926 congeners B_0 and B_1, as well as main teicoplanin congener – $A_{2\text{-}2}$ – are marked with black arrows.

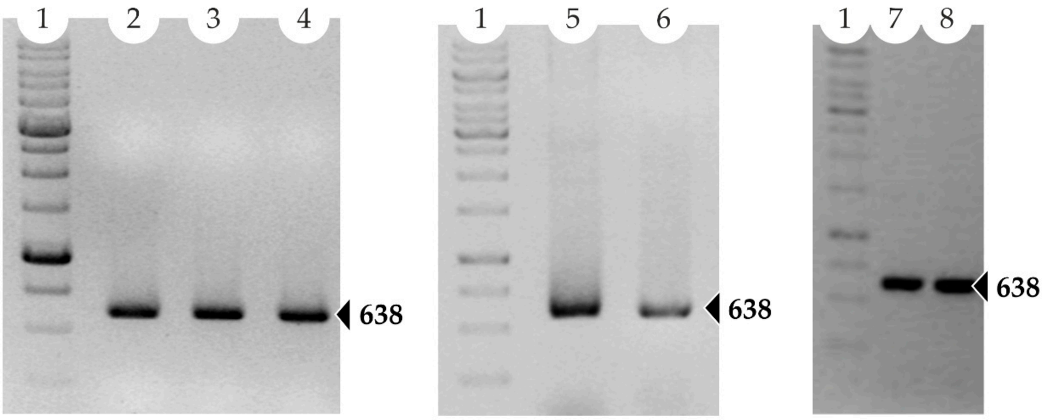

1 - GeneRuler 1 kb DNA Ladder (Thermo Fisher Scientific).
2 - pSAD3 as a template with aac(3)IV_Fw/Rv.
3 - *N. gerenzanensis* Δ*dbv3* pSAD3⁺ genomic DNA as a template with aac(3)IV_Fw/Rv.
4 - *N. gerenzanensis* Δ*dbv4* pSAD4⁺ genomic DNA as a template with aac(3)IV_Fw/Rv.
5 - pSAD4 as a template with aac(3)IV_Fw/Rv.
6 - *N. gerenzanensis* Δ*dbv4* pIJ12551*dbv4*⁺ genomic DNA as a template with aac(3)IV_Fw/Rv.
7 - pIJ12551*dbv3* as a template with aac(3)IV_Fw/Rv.
8 - *N. gerenzanensis* Δ*dbv3* pIJ12551*dbv3*⁺ genomic DNA as a template with aac(3)IV_Fw/Rv.

Figure S3. Photographs demonstrating PCR-verification of genotypes of different of complemented *N. gerenzanensis* mutants generated throughout this study. Mentioned oligonucleotide primers are given in Table 2, main text.

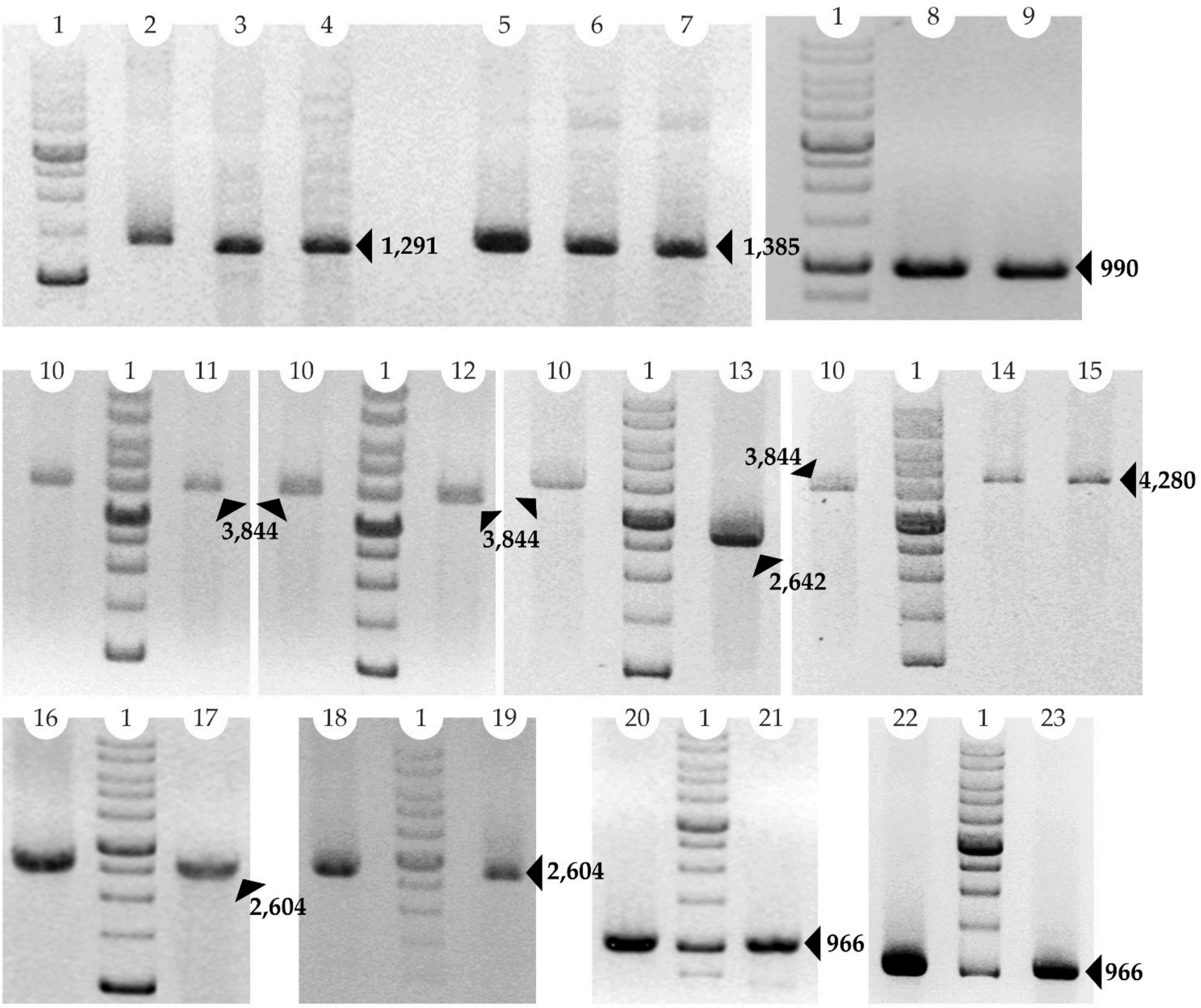

1 - GeneRuler 1 kb DNA Ladder (Thermo Fisher Scientific).
2 - pSET152Atei15* as a template with pSET_ver_F/tei15*_ver_R.
3 - *N. gerenzanensis* pSET152Atei15*$^{*+}$ genomic DNA as a template with pSET_ver_F/tei15*_ver_R.
4 - *N. gerenzanensis* Δ*dbv4* pSET152Atei15*$^{*+}$ genomic DNA as a template with pSET_ver_F/tei15*_ver_R.
5 - pSET152Atei16* as a template with pSET_ver_F/tei16*_ver_R.
6 - *N. gerenzanensis* pSET152Atei16*$^{*+}$ genomic DNA as a template with pSET_ver_F/tei16*_ver_R.
7 - *N. gerenzanensis* Δ*dbv*3 pSET152Atei16*$^{*+}$ genomic DNA as a template with pSET_ver_F/tei16*_ver_R.
8 - pIJ12551tei15* as a template with Tei15_F/RWpIJ.
9 - *N. gerenzanensis* Δ*dbv4* pIJ12551tei15*$^{*+}$ genomic DNA as a template with Tei15_F/RWpIJ.
10 - ATCC 39727 genomic DNA as a template with 3/4KOVER_F/R.
11 - *N. gerenzanensis* pSET152Atei15*$^{*+}$ genomic DNA as a template with 3/4KOVER_F/R.
12 - *N. gerenzanensis* pSET152Atei16*$^{*+}$ genomic DNA as a template with 3/4KOVER_F/R.
13 - *N. gerenzanensis* Δ*dbv*3 pSET152Atei16*$^{*+}$ genomic DNA as a template with 3/4KOVER_F/R.
14 - *N. gerenzanensis* Δ*dbv4* pSET152Atei15*$^{*+}$ genomic DNA as a template with 3/4KOVER_F/R.
15 - *N. gerenzanensis* Δ*dbv4* pIJ12551tei15*$^{*+}$ genomic DNA as a template with 3/4KOVER_F/R.
16 - pSAD3 as a template with dbv3_F/RWpIJ.
17 - *A. teichomyceticus* pSAD3^{+} genomic DNA as a template with dbv3_F/RWpIJ.
18 - pSHAD3 as a template with dbv3_F/RWpIJ.
19 - *A. teichomyceticus* Δtei16* pSHAD3^{+} genomic DNA as a template with dbv3_F/RWpIJ.
20 - pSAD4 as a template with dbv4_F/RWpIJ.
21 - *A. teichomyceticus* pSAD4^{+} genomic DNA as a template with dbv4_F/RWpIJ.
22 - pSHAD4 as a template with dbv4_F/RWpIJ.
23 - *A. teichomyceticus* Δtei15* pSHAD4^{+} genomic DNA as a template with dbv4_F/RWpIJ.

Figure S4. Photographs demonstrating PCR-verification of genotypes of different cross-complemented mutants of *N. gerenzanensis* and *A. teichomyceticus* generated throughout this study. Mentioned oligonucleotide primers are given in Table 2, main text.

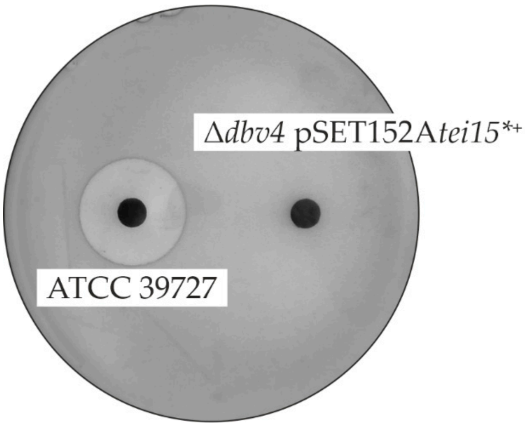

Figure S5. A40926 production in *N. gerenzanensis* Δ*dbv4* pSET152A*tei15**+ is not restored as seen from *B. subtilis* HB0933 growth inhibition assay (MH medium). To prepare the assay, culture broth samples were collected after 144 h cultivation of *N. gerenzanensis* ATCC 39727 and Δ*dbv4* pSET152A*tei15**+ in FM2 liquid medium. A40926 was extracted as reported in Materials and Methods. 50 μL of obtained extracts were loaded onto 6 mm paper disks.

(a) **(b)**

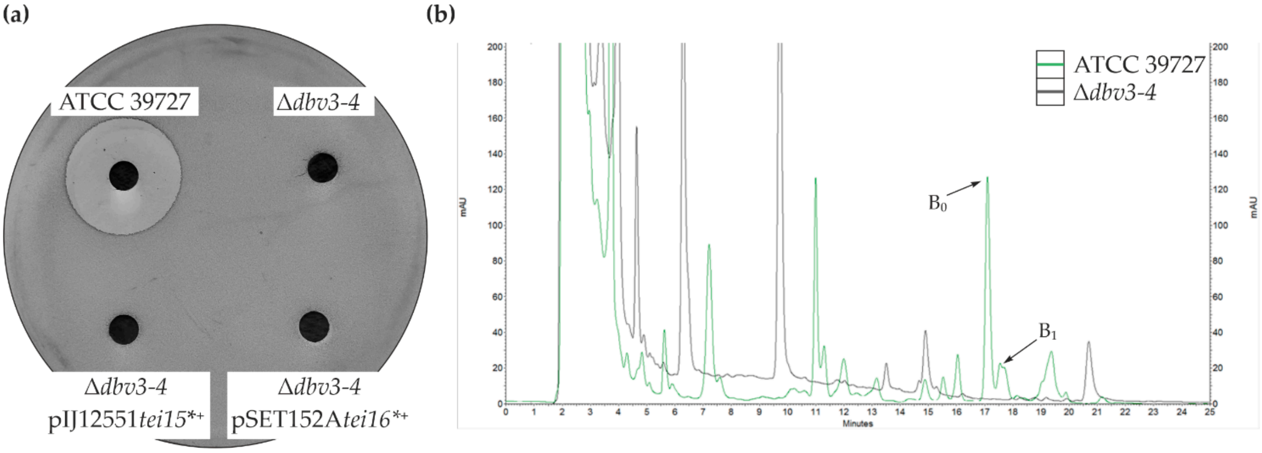

Figure S6. *N. gerenzanensis* Δ*dbv3-4* is unable to produce A40926 as demonstrated by *B. subtilis* HB0933 growth inhibition assay (MH medium) (a) and HPLC analysis (b); expression of either *tei16** or *tei15** does not activate antimicrobial activities in *N. gerenzanensis* Δ*dbv3-4* (a). a) To prepare the assay, culture broth samples were collected after 144 h cultivation of *N. gerenzanensis* ATCC 39727, Δ*dbv3-4*, Δ*dbv3-4* pIJ12551*tei15**+, and Δ*dbv3-4* pSET152A*tei16**+ strains in FM2 liquid medium. A40926 was extracted as reported in Materials and Methods. 50 μL of obtained extracts were loaded onto 6 mm paper disks. b) HPLC analyses of the extracts from *N. gerenzanensis* ATCC 39727 and Δ*dbv3-4* confirmed that Δ*dbv3-4* is unable to produce A40926 in FM2 medium; *N. gerenzanensis* ATCC 39727 chromatographic profile is shown in green, while the one for *N. gerenzanensis* Δ*dbv3-4* in black. Two main A40926 peaks could be distinguished in the *N. gerenzanensis* ATCC 39727 chromatographic profile: B_0 and B_1, indicated by the black arrows, which are absent from the chromatogram of *N. gerenzanensis* Δ*dbv3-4*.

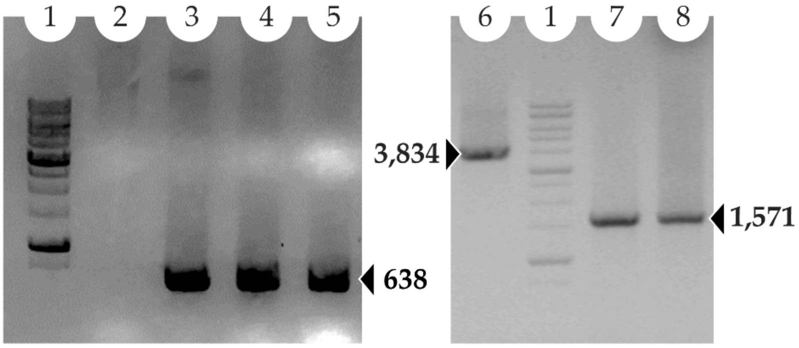

1 - GeneRuler 1 kb DNA Ladder (Thermo Fisher Scientific).
2 - *N. gerenzanensis* Δ*dbv3-4* genomic DNA as a template with aac(3)IV_Fw/Rv.
3 - pSET152A*tei16** as a template with aac(3)IV_Fw/Rv.
4 - *N. gerenzanensis* Δ*dbv3-4* pIJ12551*tei15*$^{*+}$ genomic DNA as a template with aac(3)IV_Fw/Rv.
5 - *N. gerenzanensis* Δ*dbv3-4* pSET152A*tei16*$^{*+}$ genomic DNA as a template with aac(3)IV_Fw/Rv.
6 - ATCC 39727 genomic DNA as a template with 3/4KOVER_F/R.
7 - *N. gerenzanensis* Δ*dbv3-4* pIJ12551*tei15*$^{*+}$ genomic DNA as a template with 3/4KOVER_F/R.
8 - *N. gerenzanensis* Δ*dbv3-4* pSET152A*tei16*$^{*+}$ genomic DNA as a template with 3/4KOVER_F/R.

Figure S7. Photographs demonstrating PCR-verification of genotypes of *N. gerenzanensis* and *A. teichomyceticus* strains overexpressing heterologous CSRGs generated throughout this study. Mentioned oligonucleotide primers are given in Table 2, main text.
